# Collision Tumor of the Kidney: Renal Cell Carcinoma Hidden in a Giant Angiomyolipoma in a Patient With Tuberous Sclerosis Complex

**DOI:** 10.31486/toj.20.0075

**Published:** 2021

**Authors:** José Iván Robles-Torres, José Gustavo Arrambide-Herrera, Max Molina-Ayala, Sergio Alan Dávila-Martínez, Pedro Antonio Madero-Morales, Lauro Salvador Gómez-Guerra

**Affiliations:** ^1^ Department of Urology, Hospital Universitario Dr. José Eleuterio González, Universidad Autónoma de Nuevo León, Monterrey, Nuevo León, México; ^2^ Department of Pathological Anatomy, Hospital Universitario Dr. José Eleuterio González, Universidad Autónoma de Nuevo León, Monterrey, Nuevo León, México

**Keywords:** *Angiomyolipoma*, *carcinoma–renal cell*, *tuberous sclerosis*

## Abstract

**Background:** A renal angiomyolipoma is a mixed mesenchymal benign tumor composed of smooth muscle, adipose tissue, and blood vessels. Malignant transformation of angiomyolipomas is anecdotal. To our knowledge, only 6 cases have been reported, and 4 of the patients had tuberous sclerosis complex diagnosed.

**Case Report:** We present the case of a 29-year-old male with tuberous sclerosis complex who arrived at the emergency room with gross hematuria and a painful right-sided abdominal mass. Imaging studies revealed active bleeding from a giant angiomyolipoma. An emergency nephrectomy was performed. Histopathology evaluation revealed an angiomyolipoma with a focal lesion and clear cell renal carcinoma within the tumor.

**Conclusion:** Limited evidence is available to dictate management of collision tumors of the kidney in the scenario of tuberous sclerosis complex, so a multidisciplinary approach that includes urology, oncology, genetics, and nephrology intervention needs to be considered. No standardized follow-up modality has been established for angiomyolipomas, so patients should be placed under active surveillance, similar to that carried out in cases of renal cell carcinoma.

## INTRODUCTION

Tuberous sclerosis complex (TSC)—a rare, multisystemic genetic disorder that causes benign tumors to grow in different organs, such as the brain, eyes, kidneys, heart, lungs, and skin—is the second most common neurocutaneous syndrome with a prevalence rate of 1/6,000 births.^1^ TSC is caused by mutations on 2 genes: TSC1 and TSC2.^2^ The most affected organ is the brain, resulting in clinical manifestations such as seizures, mental retardation, and skin abnormalities. The kidney is involved in approximately 80% to 85% of cases.^3^ Patients with TSC have an increased incidence of tumors, such as multiple renal angiomyolipomas, renal cell carcinoma, and oncocytoma.^4^ An angiomyolipoma is a mixed mesenchymal benign tumor composed of smooth muscle, adipose tissue, and blood vessels.^5^ Malignant transformation of these tumors is anecdotal. The coexistence of renal cell carcinoma and renal angiomyolipoma within the same tumor mass, also called collision tumor, is unusual, and, to our knowledge, only 6 cases have been reported.^3,4,6-9^ We present a case of a young male with TSC and a giant angiomyolipoma that contained a focal lesion with renal cell carcinoma.

## CASE REPORT

A 29-year-old male with a medical history of TSC diagnosed at age 16 years presented with sudden onset of gross hematuria and a painful, firm, right-sided abdominal mass. Vital signs upon arrival were temperature of 36.4 °C, blood pressure of 90/60 mmHg, respiration of 22/min, and heart rate of 110/min. Physical examination revealed generalized pallor, multiple cutaneous nodules on the face, hypopigmented skin lesions, and generalized angiofibromas. Abdominal examination showed a painful solid mass located on the right flank. Laboratory findings on arrival were hemoglobin 4.0 g/dL, leukocyte count 14.0 × 10^9^/L, C-reactive protein 4.5 mg/L, creatinine 2.3 mg/dL, and estimated glomerular filtration rate 35.9 mL/min/1.73 m^2^. Initial resuscitation with 2 L of 0.9% saline solution and 2 units of red blood cells was performed. Transurethral catheter was placed and showed gross hematuria.

**Figure 1.**
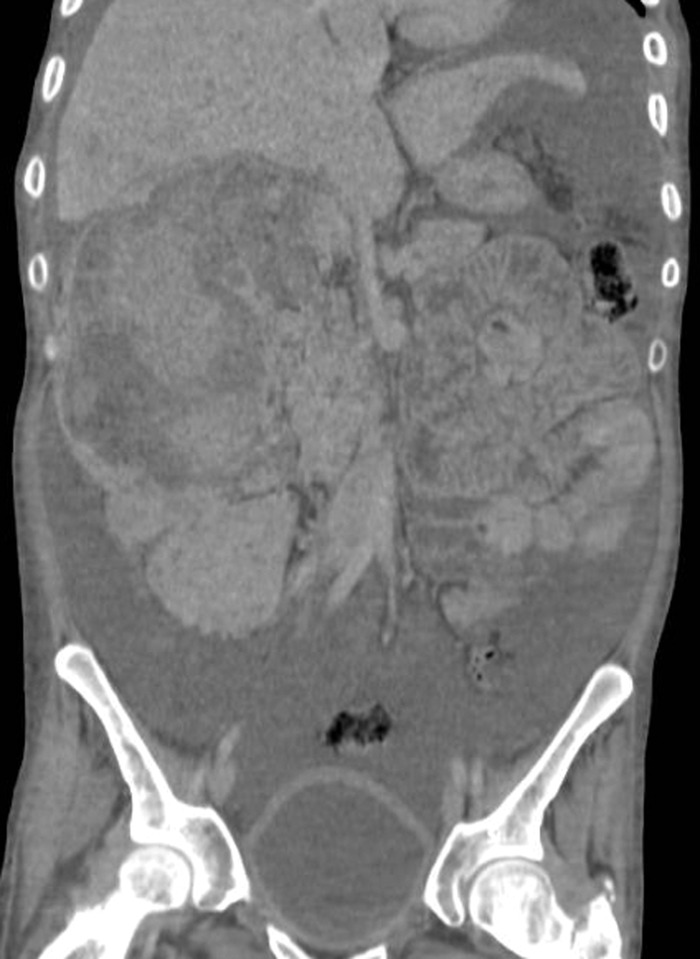
Computed tomography scan in coronal view shows a large right perinephric hematoma, left kidney with multiple angiomyolipomas, and abundant free fluid in the peritoneal cavity.

**Figure 2.**
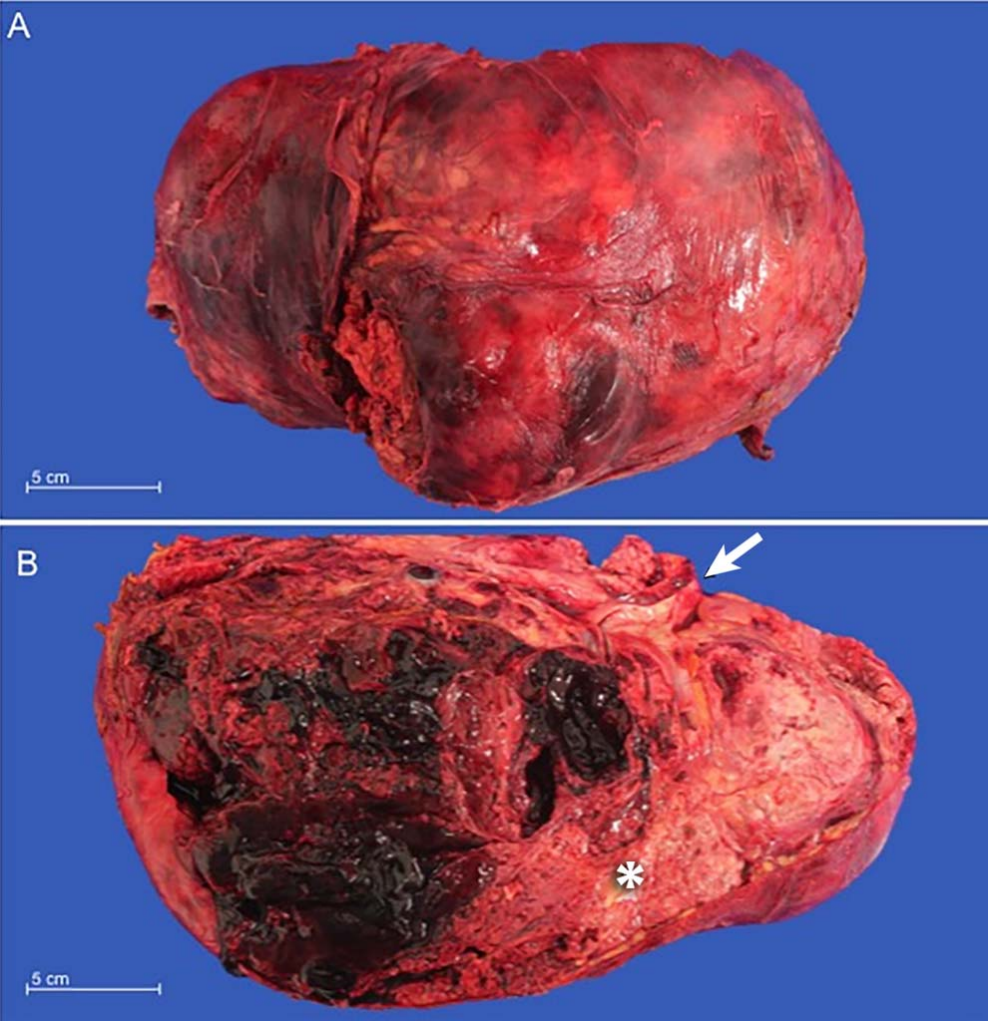
(A) Macroscopic view of the kidney. (B) Cross-section of the kidney shows visible tumor with a tan, whorled appearance and admixed fat tissue (asterisk). The ureter was identified (arrow).

**Figure 3.**
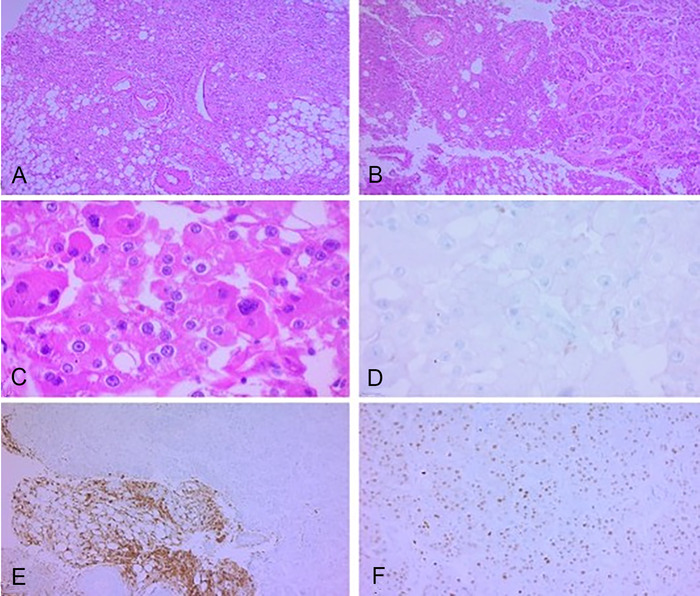
Hematoxylin and eosin stained sections of the patient's kidney. (A) The arrangement of adipocytes, spindle cells, and enlarged thick-walled vessels with irregular walls is mostly haphazard (×100). (B) A clear transition to a different tumor is observed in nests within a collagenous stroma (×100). (C) In detail (×400), pleomorphic nuclei and prominent nucleoli are observed, with a finely granular eosinophilic cytoplasm and a fine capillary network, compatible with renal cell carcinoma. The entire area with these characteristics was surrounded by angiomyolipoma. Immunohistochemistry was performed to further identify this foci, with CD117 negativity (D), HMB45 positivity only in the angiomyolipoma component (E), and PAX8 nuclear positivity in the renal cell carcinoma (F).

Contrast-enhanced computed tomography (CT) scan showed bilateral renal angiomyolipomas, with a predominant right renal mass measuring 12 × 12 × 18 cm^3^ (Figure 1) and showing signs of active arterial bleeding. A simple nephrectomy was performed. The kidney was enlarged and irregular, with heterogeneous tanning and admixed fat tissue within the tumor (Figure 2). Light microscopy showed multiple angiomyolipomas, and one section showed a well-circumscribed lesion within the giant angiomyolipoma that was composed of clear cells with finely granular eosinophilic cytoplasm and marked nuclear enlargement with prominent nucleoli (Figure 3).

Immunohistochemistry results were HMB45 positive and PAX8 negative, confirming the diagnosis of angiomyolipoma. The focal lesion had PAX8 positive and HMB45 negative cells, compatible with a renal cell carcinoma clear cell variant, and was completely surrounded by angiomyolipoma. The tumor was classified as World Health Organization/International Society of Urological Pathology (WHO/ISUP) grade 4.

During the postoperative period, the patient's clinical response was favorable, with maintenance of hemodynamic stability. However, his renal function remained affected, and he required temporary renal replacement therapy. After surgery, the patient maintained a baseline creatinine of 1.3 mg/dL and a glomerular filtration rate of 69.4 mL/min/1.73 m^2^. After a 14-day hospital stay, the patient was discharged with close follow-up.

Surveillance of the contralateral kidney with a multidisciplinary approach involving various specialties—including urology, oncology, radiology, genetics, and nephrology—was chosen because no evidence supports active treatment in this specific setting. Based on the recommendations for active surveillance for renal cell carcinoma, a CT scan was scheduled for 3 months after surgery, and then every year if no suspicious changes were detected. Renal biopsy was suggested if a suspicious lesion were detected during follow-up.^10^ After 1 year of follow-up with periodic clinical and radiologic evaluations, no evidence of recurrence has been reported in the remaining kidney.

## DISCUSSION

Coexistence of renal cell carcinoma and angiomyolipoma within the same tumor is anecdotal, with only 6 cases reported,^3,4,6-9^ and 4 of the patients had TSC.^3,4,6,8^ The mean age of the patients with collision tumors was 25.5 ± 1.2 years, similar to our case.^3,4,6-9^ Approximately 50% to 80% of patients with TSC will have angiomyolipomas.^4^ Renal angiomyolipomas are mixed mesenchymal tumors belonging to the family of perivascular epithelioid cell tumors; they are the most common kidney manifestation in TSC.^5,11^ In this scenario, angiomyolipomas are bilateral, multiple, and usually asymptomatic. In some cases, angiomyolipomas can have an accelerated growth rate, causing pain, kidney failure, or sudden bleeding.^11^

Even in cases with cytologic atypia or lymph node involvement, angiomyolipomas are considered benign. In cases of transformation to renal cell carcinoma variants, the initial morphology mandates are supported with immunohistochemistry. Angiomyolipomas are positive for melanocytic tumor markers, such as HMB45 and Melan-A, and can also be expressed in mature fat tissue and blood vessels.^12^ Granular eosinophilic cytoplasm warrants the differential diagnosis of oncocytoma, clear cell, and chromophobe variants of renal cell carcinoma. Some of the most important and useful markers for differential diagnosis of renal cell carcinoma are cytokeratins, vimentin, PAX2, and PAX8. Renal cell carcinoma is negative for melanocytic tumor markers.^12^

No standardized follow-up has been established for angiomyolipomas. Indications for further imaging studies are angiomyolipomas >4 cm, recurrent hematuria, and pain related to tumor growth. Once renal cell carcinoma is confirmed after nephrectomy, a CT scan must be performed 3 months after surgery and then annually for 3 years or longer as clinically indicated. A more rigorous imaging schedule can be considered if positive margins or adverse pathologic features are reported, such as sarcomatoid morphology or high grade (3 or 4) according to the WHO/ISUP staging system.^10^

## CONCLUSION

Malignant transformation of angiomyolipomas is extremely rare and limited to anecdotal reports. Most of the patients reported with these findings have TSC. Because of limited evidence, an etiologic cause of this condition cannot be established. Active surveillance is recommended in patients with TSC and angiomyolipomas, similar to the surveillance recommended for renal cell carcinoma, with the aim of detecting early changes suggestive of malignant transformation or complications related to tumor growth.

## Notes

*This article meets the Accreditation Council for Graduate Medical Education and the American Board of Medical Specialties Maintenance of Certification competencies for Patient Care and Medical Knowledge.*
